# Simultaneous targeting of PI3Kδ and a PI3Kδ-dependent MEK1/2-Erk1/2 pathway for therapy in pediatric B-cell acute lymphoblastic leukemia

**DOI:** 10.18632/oncotarget.2533

**Published:** 2014-09-26

**Authors:** Xiang Wang, Xi Zhang, Ben-shang Li, Xiaowen Zhai, Zhuo Yang, Li-xia Ding, Hongsheng Wang, Chris Liang, Weiliang Zhu, Jian Ding, Ling-hua Meng

**Affiliations:** ^1^ Division of Anti-Tumor Pharmacology, State Key Laboratory of Drug Research, Shanghai Institute of Materia Medica, Chinese Academy of Sciences, Shanghai, China; ^2^ Drug Discovery and Design Center, State Key Laboratory of Drug Research, Shanghai Institute of Materia Medica, Chinese Academy of Sciences, Shanghai, China; ^3^ Department of Hematology and Oncology, Shanghai Jiaotong University School of Medicine, Shanghai, China; ^4^ Department of Hematology and Oncology, Children's Hospital of Fudan University, Shanghai, China; ^5^ Xcovery, LLC, West Palm Beach, Florida, USA

**Keywords:** PI-3Kdelta inhibitor, MAPK, B cell acute lymphocytic leukemia, target therapy

## Abstract

B cell acute lymphoblastic leukemia (B-ALL) is the most common hematological malignancy diagnosed in children, and blockade of the abnormally activated PI3Kδ displayed promising outcomes in B cell acute or chronic leukemias, but the mechanisms are not well understood. Here we report a novel PI3Kδ selective inhibitor X-370, which displays distinct binding mode with p110δ and blocks constitutively active or stimulus-induced PI3Kδ signaling. X-370 significantly inhibited survival of human B cell leukemia cells in vitro, with associated induction of G1 phase arrest and apoptosis. X-370 abrogated both Akt and Erk1/2 signaling via blockade of PDK1 binding to and/or phosphorylation of MEK1/2. Forced expression of a constitutively active MEK1 attenuated the antiproliferative activity of X-370. X-370 preferentially inhibited the survival of primary pediatric B-ALL cells displaying PI3Kδ-dependent Erk1/2 phosphorylation, while combined inhibition of PI3Kδ and MEK1/2 displayed enhanced activity. We conclude that PI3Kδ inhibition led to abrogation of both Akt and Erk1/2 signaling via a novel PI3K-PDK1/MEK1/2-Erk1/2 signaling cascade, which contributed to its efficacy against B-ALL. These findings support the rationale for clinical testing of PI3Kδ inhibitors in pediatric B-ALL and provide insights needed to optimize the therapeutic strategy.

## INTRODUCTION

B-cell acute lymphoblastic leukemia (B-ALL) is predominantly a childhood disease with approximately 75% of patients younger than 6 years of age [1-4]. A better understanding of the mechanisms of drug response/resistance are still urgently required in order to further improve the outcomes and quality of life associated with current treatment as well as to develop innovative targeted therapeutics [5]. Phosphoinositide 3-kinases (PI3Ks) are a family of lipid kinases critically involved in a variety of cellular functions including growth, proliferation, differentiation, motility, survival and intracellular trafficking [6, 7]. The PI3K pathway is frequently deregulated in a wide range of tumor types as a result of genetic and epigenetic aberrations [8-10]. PI3Kδ, which is largely enriched in the hematopoietic system, is hyper-activated in most B-cell lymphoblastic leukemia and has attracted increasing interest as a target for therapy in certain leukemias [10, 11]. The development of idelalisib (GS-1101, CAL-101) [12-14], a PI3Kδ-specific inhibitor that was approved recently to treat patients with relapsed follicular B-cell non-Hodgkin lymphoma (FL) and relapsed small lymphocytic lymphoma (SLL), another type of non-Hodgkin lymphoma, has validated PI3Kδ as a promising target for adult B-cell malignancies. However, the efficacy and mechanism(s) of action of PI3Kδ inhibitors in childhood B-ALL remains unclear.

The well-studied PI3K/Akt and Ras/MAPK cascades influence each other at multiple nodes and phases of signal propagation in both negative and positive manners [15-19], resulting in dynamic and complex crosstalk in normal and tumor cells [20-22]. It has been reported that CAL-101 inhibits phosphorylation of both Akt and Erk1/2 in multiple myeloma cells, suggesting a PI3Kδ-dependent mechanism driving Erk1/2 signaling [23]. However, the molecular mechanisms underlining the processes and its clinical relevance have yet to be elucidated.

Though CAL-101 has been demonstrated to be successful for the treatment of B-cell malignancies, new PI3Kδ inhibitors with different binding modes are likely needed to mitigate the resistance which invariably develops against single kinase inhibitor. Here, we introduce X-370, a novel PI3Kδ selective inhibitor which displays a different binding mode to PI3Kδ compared to CAL-101. Its potency against B-ALL was evaluated in cell lines and primary cells. Notably we report that inhibition of PI3Kδ with X-370 leads to abrogation not only of PI3K signaling but also of Erk1/2 signaling via an atypical PI3K-PDK1-MEK1/2-Erk1/2 signaling cascade, which we describe for the first time in this report. Inhibition of PI3Kδ-dependent Erk1/2 phosphorylation by PI3Kδ inhibitor serves as an efficient marker of its efficacy against childhood B-ALL since simultaneous targeting PI3Kδ and MEK1/2 may further improve the efficacy of PI3kδ inhibition.

## RESULTS

### X-370 is a potent selective PI3Kδ inhibitor

**Figure 1:**
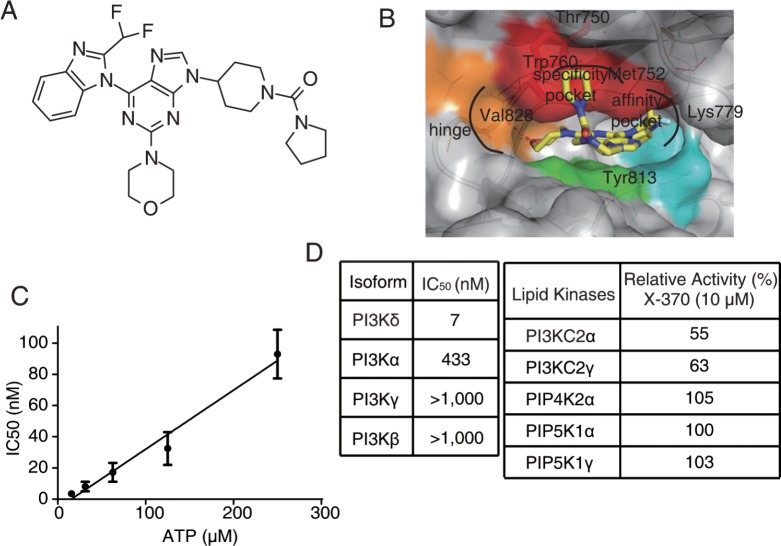
Structure of X-370 and its inhibitory activity against lipid kinases (A) Chemical structure of X-370. (B) X-370 docked to the crystal structure of PI3Kδ. The model was generated based on the co-crystal structure of PI3Kδ and ZSTK474 (PDB ID: 2WXL). (C) IC_50_ of X-370 against PI3Kδ increased along with ATP concentration. (D) X-370 displayed high selective activity against PI3Kδ isoforms among representative lipid kinases. Data was obtained from Kinase Profiler Services (Millipore, United Kingdom). Briefly, X-370 was solved in DMSO at a concentration of 10 mM, 10-point kinase inhibitory activity was measured over concentrations in a half-log dilution series started at 1000 nM with ATP at a concentration consistent with Km of each isoform of class I PI3K and IC_50_s were calculated. Relative kinase activity in the presence of 10 μM X-370 compared to DMSO control was measured for the rest of lipid kinases.

Utilizing the crystal structure of PI3Kδ, we designed and synthesized a series of compounds in an effort to discover new potent and selective PI3Kδ inhibitors, which bind to PI3Kδ differently from CAL-101. As X-370 displayed the most potent activity against PI3Kδ in a pilot screening, it was selected for further investigation. X-370, which possesses a difluromethyl benzoimidazole and morpholino moiety similar to ZSTK474 (Figure 1A), was docked into PI3Kδ based on the co-crystal structure of PI3Kδ and ZSTK474 (PDB ID: 2WXL) [24]. As shown in Figure 1B, the morpholino ring of X-370 adopts a chair conformation and the oxygen of the morpholino groups is positioned as the hinge hydrogen bond acceptor for the backbone Val828. The benzimidazole group of X-370 extends into the affinity pocket, where its nitrogen acts as a hydrogen bond acceptor for the primary amine of Lys779. The difluoromethyl group of X-370 points toward Pro758 in the hydrophobic affinity pocket. Instead of wedging between Met752 and Trp760, a binding mode utilized by CAL-101 to generate specificity against PI3Kδ (Figure S1), the pyrrole group of X-370 presses tightly against Trp760. These specific interactions of X-370 with p110δ confer a selective and potent inhibition of PI3Kδ. As expected, X-370 inhibited the kinase activity of PI3Kδ in an ATP-competitive manner, with IC_50_ increasing in concert with ATP levels (Figure 1C), confirming the binding of X-370 in the ATP pocket. As shown in Figure 1D, X-370 significantly inhibited the kinase activity of PI3Kδ with an IC_50_ of 7 nM, which is much lower than its IC_50_s against other class I PI3K enzymes. More than 1000-fold selectivity was seen against other lipid kinases tested including PI3KC2α, PI3KC2γ, PIP4K2α, PIP5K1α and PIP5K1γ. We next tested the activity of X-370 in a panel of 61 protein kinases selected among human kinome, which represent all known kinase families. As shown in Table S1, X-370 possessed little activity against all the kinases tested at the concentration of 10 μM.

**Figure 2:**
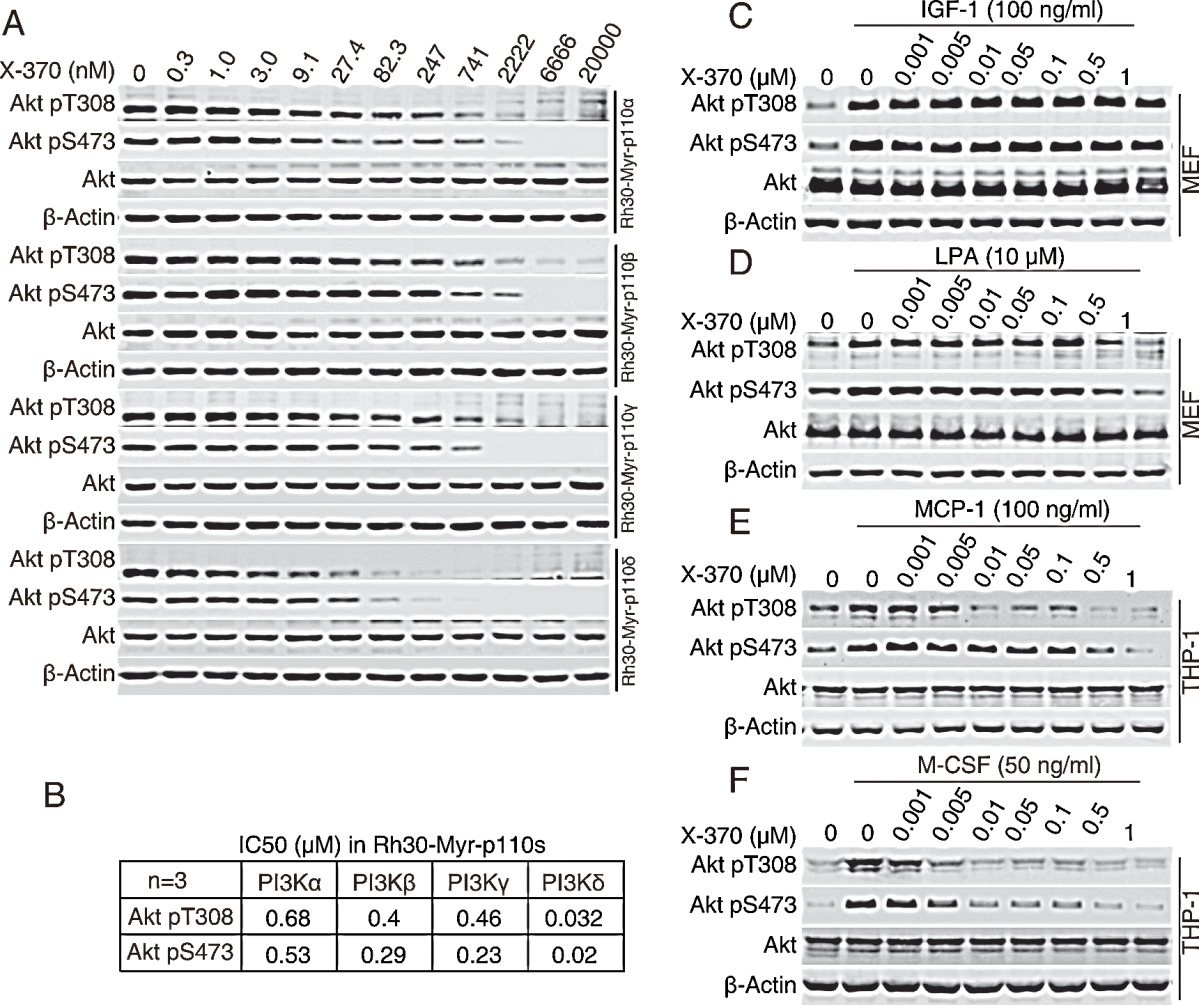
X-370 is highly selective against PI3Kδ-mediated signaling at cellular level (A) The panel of isogenic Rh30-Myr-p110α, Rh30-Myr-p110β, Rh30-Myr-p110γ and Rh30-Myr-p110δ cells were cultured in serum-free medium for 12 h and then incubated with serially diluted X-370 for an additional 1 h. Cell lysates were probed with indicated antibodies. The IC_50_ values were calculated based on relative band intensity measured with ImageQuant™ TL (GE Healthcare Life Sciences, Piscataway, NJ) (B). Data shown are mean values from three independent experiments. (C, D, E, F) X-370 blocked stimuli-triggered PI3Kδ activation. MEF cells and THP-1 were starved in serum-free medium for 12 h or 4 h respectively and X-370 were added for an additional 1 h treatment following stimulation of IGF-1 at 100 ng/ml for MEF cells (C), LPA at 10 μM for MEF cells (D), MCP-1 at 100ng/ml for THP-1 (E) and M-CSF at 50 ng/ml for THP-1 (F). Similar results were derived from 3 independent experiments.

To assess the selectivity of X-370 in the context of an isogenic cellular background, we generated a panel of Rh30 human rhabdomyosarcoma cell lines each of which stably expresses a single myristoylated (Myr)-epitope tagged p110 human class I PI3K catalytic subunit, termed as Rh30-Myr-p110α, Rh30-Myr-p110β, Rh30-Myr-p110γ or Rh30-Myr-p110δ. In these cells, endogenous PI3K is not activated under serum-free culture conditions, whereas the ectopically expressed p110 isoforms are membrane-anchored and constitutively active due to myristoylation at N-terminal. Utilizing this cell panel, isoform-specific PI3K activity can be determined for any given inhibitor[25]. These cells were treated with X-370 and phosphorylated Akt was detected as the readout of PI3K activity. X-370 inhibited phosphorylation of Akt at both Thr308 and Ser473 in a dose-dependent manner in four tested cell lines (Figure 2A). Quantitative analysis revealed that X-370 preferentially blocked Akt phosphorylation in Rh30-Myr-p110δ cells with an EC_50_ of 32 nM (Akt pT308) or 20 nM (Akt pS473), which is at least 10 fold lower than in isogenic Rh30-Myr-p110α/β/γ cells (Figure 2B). Similar property was also observed in CAL-101 (Figure S2).

In fibroblasts, signals from the insulin-like growth factor 1 (IGF-1) receptor are mediated by PI3Kα, while lysophosphatidic acid (LPA) stimulates G-protein coupled receptor (GPCR) signals via PI3Kβ[26]. After being pre-incubated with X-370, murine embryonic fibroblasts cultured in serum-free medium were stimulated with IGF-1 or LPA. X-370 partially inhibited IGF-1- or LPA-induced Akt phosphorylation up to 0.5 μM (Figure 2C & D). Either PI3Kδ or PI3Kγ can be activated in THP-1 monocytes dependent on the the stimulating ligand, among which macrophage colony stimulating factor (M-CSF/CSF-1) activates RTK-coupled PI3Kδ, while monocyte chemoattractant protein-1 (MCP-1) activates PI3Kγ [24]. Accordingly, THP-1 cells cultured in serum-free medium were stimulated with MCP-1 or M-CSF in the presence of X-370 and phosphorylated Akt was measured. As shown in Figure 2E & F, X-370 completely blocked M-CSF-induced Akt phosphorylation at 10 nM, while it displayed much less activity against MCP-1-stimulated Akt phosphorylation.

Thus, X-370 is a novel potent PI3Kδ inhibitor and possesses high selectivity for PI3Kδ relative to other class I PI3K isoforms at both molecular and cellular levels.

### PI3Kδ inhibition led to blockade of B cell leukemia cells, proliferation *in vitro*

**Figure 3:**
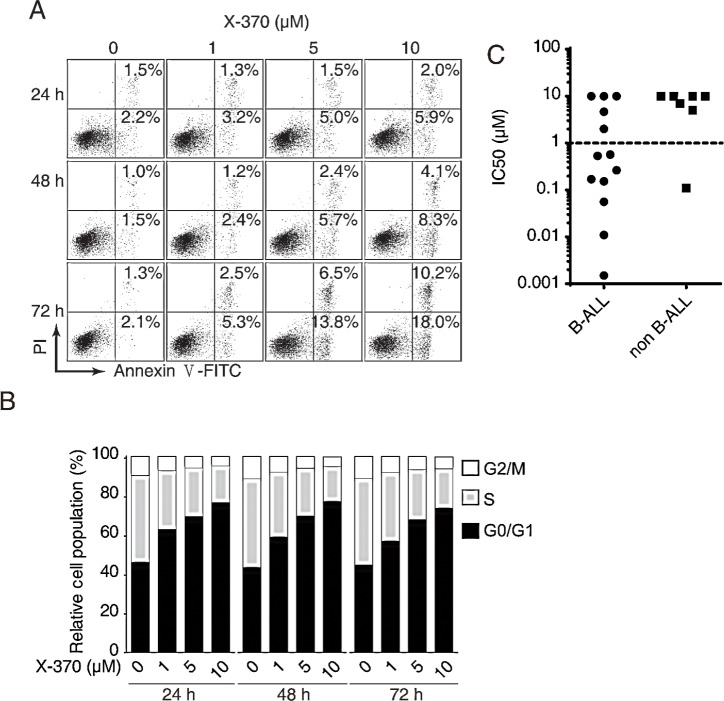
PI3Kδ inhibition led to blockade of B cell leukemia cells proliferation via induction of cell cycle arrest and apoptosis Raji cells treated with X-370 for indicated times and at serial diluted concentrations were subjected to Annexin V-FITC/PI staining (A) and PI staining (B) and FACS analysis to show cell apoptosis and cell cycle distribution respectively. (C) X-370 inhibited proliferation of human leukemia primary cells. Freshly isolated human primary mononuclear cells from bone marrow samples of patients were treated with X-370 for 72 h and cell viability was assayed using CCK-8 assay. IC_50_s of each sample were plotted where 10 μM was assigned to specimens with IC_50_ >10 μM. Each symbol represents an individual patient sample.

PI3Kδ plays an important role in B cell survival and regulates the G1/S transition in the cell cycle. We found that X-370 potently inhibited proliferation of a panel of human leukemia cells (Figure S3). To better understand the function of PI3Kδ in human B cell lymphoblastic leukemia cells, we measured apoptosis induced by X-370 in Raji cells. Annexin V-positive cells increased in time- and dose-dependent manners (Figure 3A). However, relatively higher concentrations and long term treatment were required for X-370 to induce apoptosis, as apoptotic cells were detectable only with ≥5 μM of X-370 for 72 h. Similarly, caspase 3/7 activity increased in the presence of higher concentrations of X-370 (≥5 μM) (Figure S4). Since only about 28% cells underwent apoptosis in the presence of 10 μM X-370 for 72 h, apoptosis cannot explain the potent activity of X-370 against cell proliferation. We next evaluated the effect of X-370 on cell cycle distribution. As shown in Figure 3B, treatment with X-370 for 24 h led to increase in cell population in G1 phase, which was accompanied with reduced cell population in S phase. More cells accumulated in G1 phase arrest with increasing concentrations of X-370, however longer treatment failed to further enhance this effect. DNA synthesis was readily inhibited after treatment of 1 μM X-370 for 24 h and further decreased with higher concentration of X-370 (Figure S4). These results suggest that inhibition of PI3Kδ by X-370 resulted in blockade of DNA synthesis and cell cycle progression, but higher concentration and longer treatment are required to induce apoptosis, which might be resulted from inhibition of other class I PI3K isoforms by X-370. Similar phenomena were observed with CAL-101[12].

To assess the potency of X-370 in blocking B-ALL cells in more clinically relevant context, we separated primary mononuclear cells from bone marrows of diagnosed leukemia pediatric patients. Freshly prepared cells were incubated with X-370 for 72 h and cell viability was assessed. 61% (8/13) and 14% (1/7) of the B-ALL and non B-ALL specimens displayed sensitivity to X-370 with IC_50_<1 μM respectively (Figure 3C). The high incidence of activity in primary B-ALL cells suggests that X-370 is a promising candidate for the therapy of B-ALL patients.

### Inhibition of both Akt and Erk1/2 phosphorylation is required for the anti-proliferative activity of X-370

**Figure 4:**
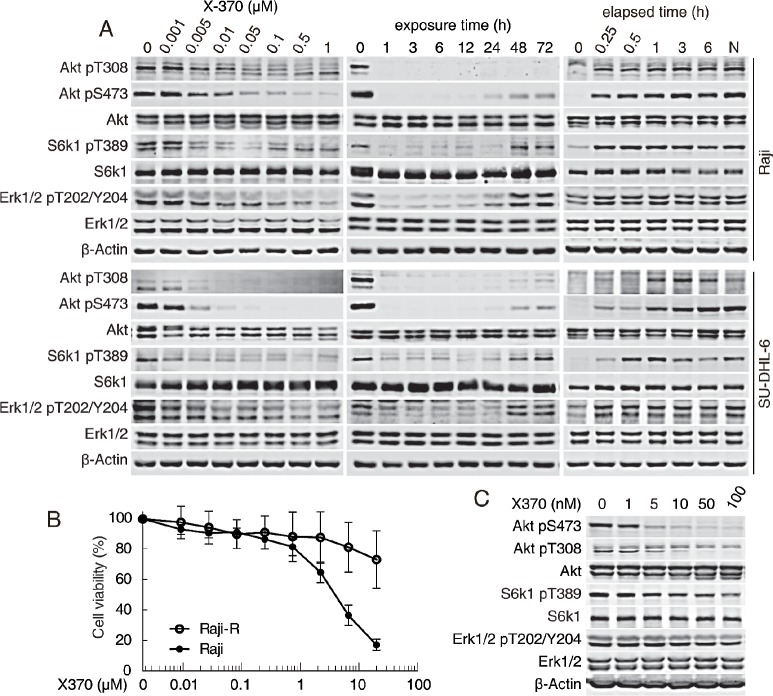
X-370 inhibited both Akt and Erk1/2 signaling in Raji and SU-DHL-6 cells (A) X-370 concentration- and time-dependently inhibited the phosphorylation of Akt and Erk1/2. Raji and SU-DHL-6 cells were treated with X-370 for 1 h (left panel) or treated with 0.5 μM X-370 for indicated times (middle panel). Right panel, Raji cells were incubated in fresh medium following treatment with 0.5 μM X-370 for 1 h. Indicated proteins were detected. (B) Raji-R (the rituximab-resistant derivatives of Raji cells) cells were resistant to X-370. The activity of X-370 on the proliferation of Raji parent and Raji-R cells was determined by CCK-8 assay as described in the methods section. (C) X-370 blocked phosphorylation of Akt but not Erk1/2 in Raji-R cells. Raji-R cells were treated with X-370 for 1h and indicated proteins were detected. Data shown are representative from three independent experiments.

We noticed that about one third of the specimens were insensitive to X-370 and wished to gain mechanistic insight underlying this differential response by dissecting the signaling in B-cell leukemia cells upon X-370 treatment. The Raji and SU-DHL-6 B-cell leukemia (or lymphoma) cells showed constitutive activation of Akt when cultured in media in the presence or absence of serum (Figure S5). Treatment of Raji and SU-DHL-6 cells with X-370 resulted in a concentration-dependent reduction in phosphorylation of both Akt (at S473 and T308) and its downstream substrates FoxO1 (at S256) and GSK-3β (at S9) as well as the mTOR target S6k1 (at T389) (Figure 4). Significantly reduction of Akt phosphorylation could be observed at X-370 concentration as low as 5 nM, which is similar to the concentration required to inhibit the biochemical activity of PI3Kδ, demonstrating a major role for PI3Kδ in constitutive PI3K signaling. However, we also found that phosphorylation of Erk1/2 at T202/Y204 decreased in lockstep with inactivation of Akt. Both signaling cascades responded quickly, within 1-h treatment, and inhibition of Akt phosphorylation at S743 and T308 persisted up to 72 h, whereas phosphorylation of S6k1 at T389 and Erk1/2 at T202/Y204 started to recover after 48-h treatment. It should be noted that Erk1/2 is not a classical downstream target of PI3K signaling, though sporadic studies have reported that inhibition of PI3K might lead to inactivation of Erk1/2 in specific cell types [23].

To test whether the effect of X-370 on PI3K signaling is reversible, Raji and SU-DHL-6 cells were incubated in drug-free medium following treatment with 0.5 μM X-370 for 1 h. As shown in Figure 4A, restoration of AKT and Erk1/2 phosphorylation could be observed after 15 minutes to 1 h upon compound removal, indicating the action of X-370 on PI3K and Erk1/2 is rapidly reversible, and suggesting that continuous inhibition of PI3K may be required to maintain efficacy.

To detect whether inhibition of Akt and Erk1/2 signaling by X-370 contribute to its antiproliferative activity, we tested the activity of X-370 in Raji-R cells, which were selected from Raji cells grown in the presence of stepwise increasing concentrations of rituximab and were resistant to rituximab [27, 28]. We found Raji-R cells were also resistant to X-370 compared to parental cells (Figure 4B). Though X-370 significantly inhibited Akt and S6k1 phosphorylation in Raji-R cells, it displayed negligible activity on the level of phosphorylated Erk1/2 (Figure 4C), suggesting simultaneous inhibition of Akt and Erk1/2 may be required for X-370 to exert its activity.

### Inhibition of PI3Kδ-PDK1-MEK1/2-Erk1/2 cascade by X-370 contributed to its antiproliferative activity

It has been reported that CAL-101 blocks Erk1/2 phosphorylation in INA-6 human myeloma cells [23], which is consistent with our results with X-370 (Figure 3) and CAL101 in Raji and SU-DHL-6 cells (Figure S6), suggesting that PI3Kδ acts upstream of Erk1/2 in some cell types. Though wild type Ras was reported to be involved in this process in breast cancer cells recently[29], any insight mechanism underlying this effect of p110δ inhibition on Erk1/2 signaling has remained obscure.

**Figure 5:**
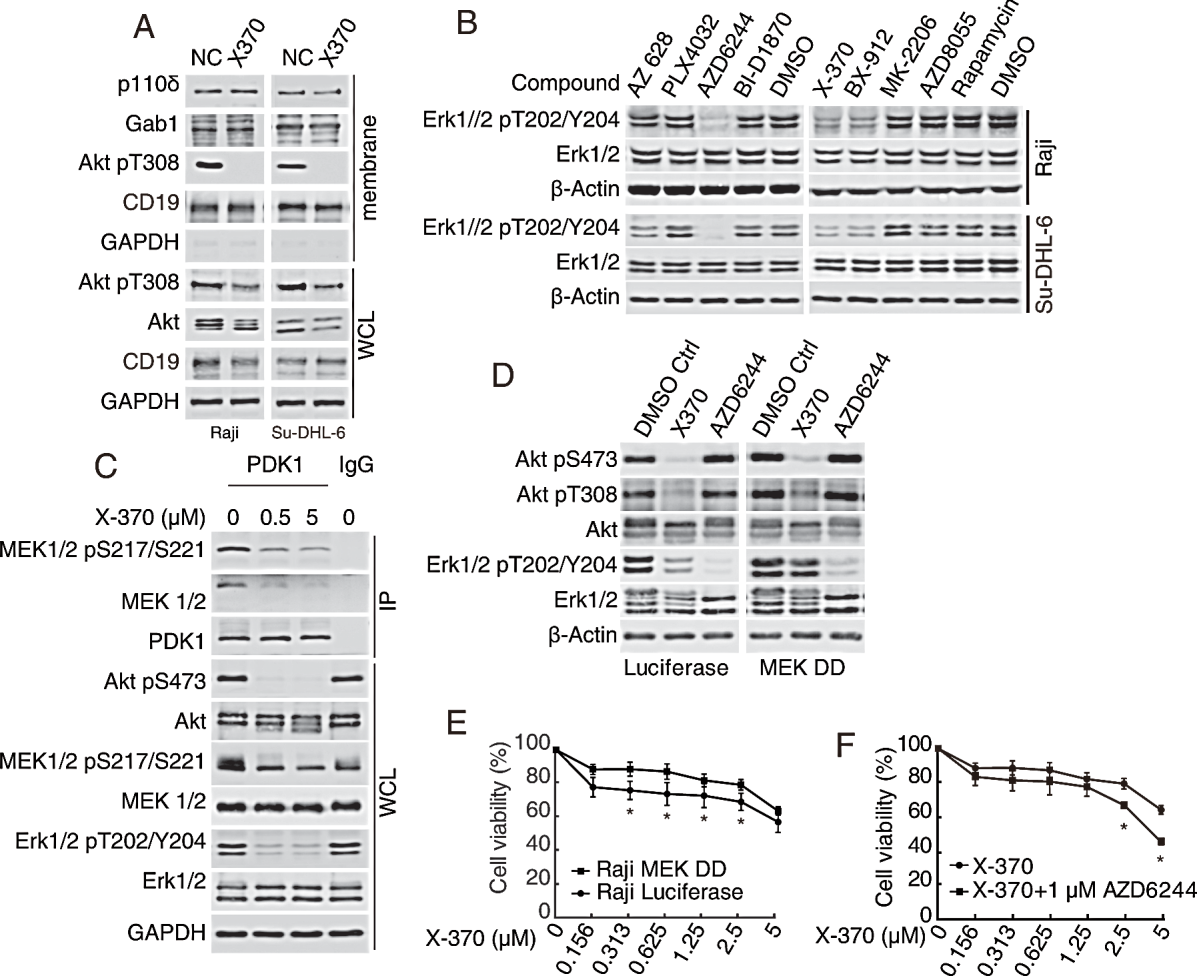
Inhibition of Erk1/2 phosphorylation by X-370 contributed to its antiproliferative activity (A-C) X-370 inhibited Erk1/2 phosphorylation via PI3K-PDK1-MEK1/2 cascade but not the PI3K-Gab1 positive feedback loop. (A) Raji and SU-DHL-6 cells were exposed to 1 μM X370 for 3 h and protein fraction from cellular membrane was separated using Native Membrane Protein Extraction Kit and probed. WCL: whole cell lysate. (B) Inhibition of PDK1 or MEK1/2 abrogated Erk1/2 phosphorylation. Cells were treated with pharmacological inhibitors of Raf (AZ 628, PLX4032), MEK1/2 (AZD6244), p90RSK (BI-D1870), PI3Kδ (X-370), PDK1 (BX-912), Akt (MK-2206), mTOR (AZD8055) or mTORC1 (Rapamycin) respectively and indicated proteins were detected. (C) X-370 disrupted the association between PDK1 and MEK1/2. Raji cells were treated with 1 μM X370 for 1 h and cell lysates were immunoprecipitated with anti-PDK1 antibody and indicated proteins were detected. WCL: whole cell lysate. (D-F) Inhibition of Erk1/2 phosphorylation was required for the anti-proliferative activity of X-370. (D) Raji cells ectopically expressing a constitutively activated phospho-mimic MEK1 mutant (MEK1 S202D/S204D or MEK DD) were incubated with X-370 or AZD6244 for 1 h and then subjected to Western blot. (E) Raji and Raji MEK DD cells were treated with X-370 for 72 h and cell proliferation was detected by CCK-8 assay. (F) Raji MEK DD cells were treated with X-370 and AZD6244 and cell proliferation was detected by CCK-8 assay. Data were show as mean ± SD of three independent experiments. *: p < 0.05 determined by t-tests at each data point.

PIP3 embedded in cellular membranes recruits the PH-domain containing protein Gab1, which in turn recruits PI3K and at the same time binds to Shp2 (Src homology 2 domain-containing protein tyrosine phosphatase) to promote the activation of Ras-Raf-MEK1/2-Erk1/2 pathway[30-32]. Recruitment of Gab1 to cellular membranes by PIP3 facilitates the formation of a positive feedback loop to amplify MAPK and PI3K signaling. Since X-370 is unlikely to directly inhibit kinases in Ras-Raf-MEK1/2 pathway (Table S1), we investigated whether inhibition of Erk1/2 phosphorylation by X-370 was due to disruption of this positive feedback. As shown in Figure 5A, the protein levels of p110δ and Gab1 in the cellular membrane fraction remained unchanged after X-370 treatment. By contrast, X-370 abolished phosphorylation of Akt at the membrane. These results indicate that PIP3 is not required to recruit Gab1 in this circumstance and that inhibition of Erk1/2 phosphorylation by X-370 is not due to disruption of Gab1-Ras-Raf-MEK1/2-Erk1/2 cascade.

We next decided to dissect the nodal protein(s) in the canonical Raf-MEK1/2-Erk1/2-RSK and PI3K-Akt-mTOR pathways contributing to inhibition of Erk1/2 phosphorylation by X-370 using a panel of pharmacological inhibitors to interrogate the key pathway components. Inhibitors of Raf (AZ 628, PLX4032), MEK1/2 (AZD6244), p90RSK (BI-D1870), PI3Kδ (X-370), PDK1 (BX-912), Akt (MK-2206), mTOR (AZD8055) and mTORC1 (Rapamycin) were employed to treat Raji and SU-DHL-6 cells. As shown in Figure 5B, apart from X-370, the MEK1/2 inhibitor AZD6244 down-regulated phosphorylated Erk1/2. Surprisingly, the PDK1 inhibitor BX-912 displayed the same effect. Notably, treatment with X-370 also inhibited phosphorylation of MEK1/2 at Ser217/221 (Figure 5C). These results implicate phosphorylation of MEK1/2 by PDK1 in a PI3Kδ-dependent way. We further found that inhibition of PI3Kδ by X-370 led to dissociation of MEK1/2 and PDK1, which was accompanied with decreased phosphorylation of both MEK1/2 and Erk1/2 (Figure 5C). Though it has been reported that PDK1 phosphorylates MEK1/2 biochemically *in vitro* and in cells independent of PI3K [33], our results strongly suggest that PI3K plays a positive role in PDK1-mediated phosphorylation of MEK1/2 and its substrates Erk1/2 in Raji cells.

As Erk1/2 acts downstream of PI3Kδ in Raji cells, its potential contribution to PI3Kδ-mediated cell viability was tested. X-370 failed to inhibit Erk1/2 phosphorylation in Raji cells ectopically expressing a constitutively activated phospho-mimic MEK1 mutant (MEK1 S202D/S204D or MEK DD), while AZD6244 abolished this process in both MEK1 mutant and wild type cells (Figure 5D). Accordingly, MEK DD expression attenuated inhibition of viability by X-370 in Raji cells (Figure 5E), while AZD6244 enhanced the activity of X-370 against Raji cells expressing MEK DD (Figure 5F), even though AZD6244 alone had little activity against both Raji cell lines (Figure S7).

### X-370 preferentially inhibited the survival of primary B-ALL cells exhibiting PI3Kδ-dependent Erk1/2 phosphorylation, while its combination with AZD6244 possessed enhanced potency

**Figure 6:**
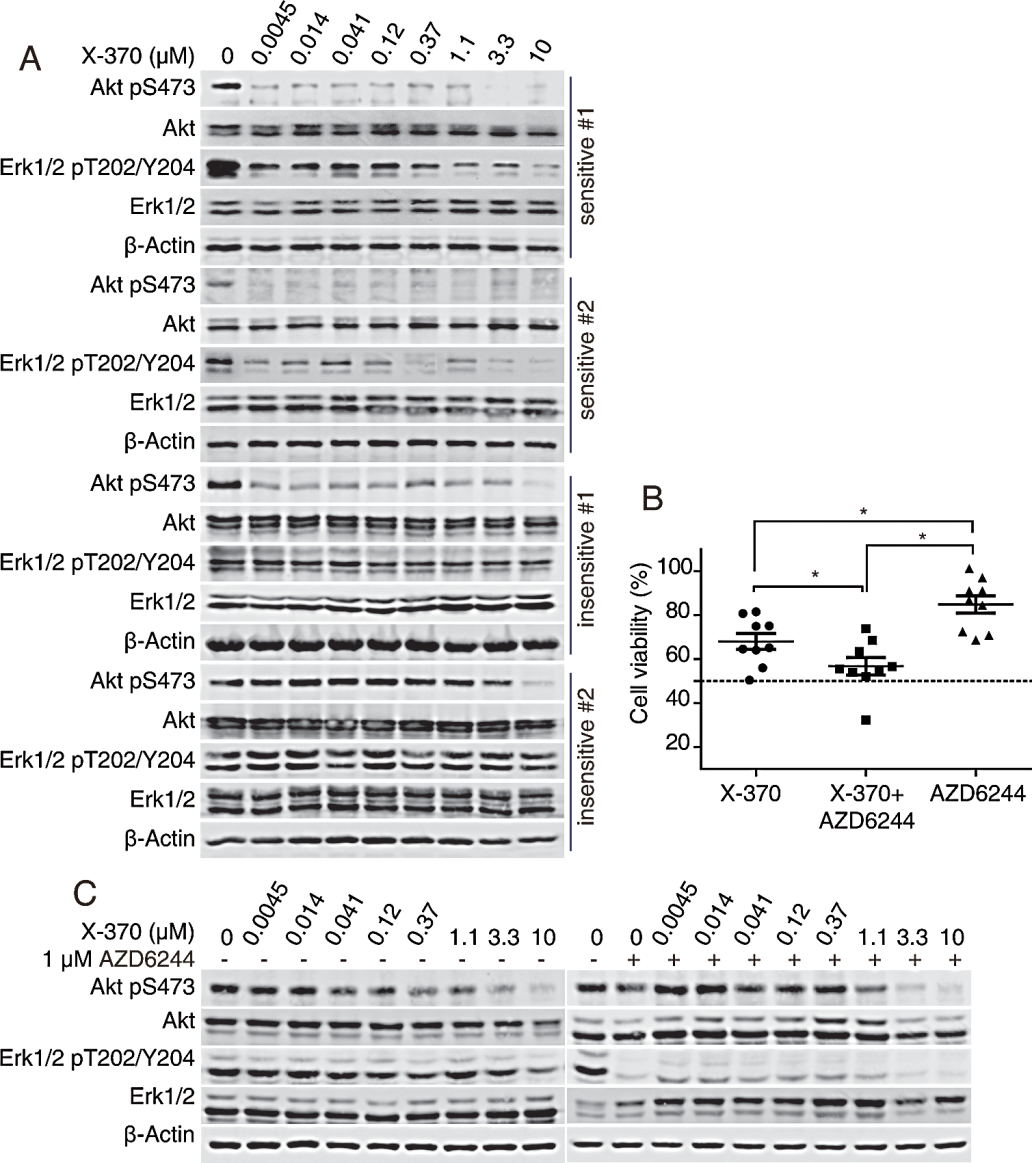
X-370-sensitive human primary B-ALL cells contained PI3K-dependent Erk1/2 phosphorylation and combination of AZD6244 and X-370 enhanced inhibitory activity against resistant specimens (A) X-370-sensitive human primary B-ALL cells contained PI3K-dependent Erk1/2 phosphorylation. Primary B-ALL cells were treated with series diluted X-370 for 72 h. Phosphorylation of Akt and Erk1/2 were detected. (B). Combination of AZD6244 and X-370 enhanced inhibitory activity against resistant specimens. X-370 resistant primary B-ALL cells were treated with 1 μM X-370 alone or cocurently with MEK1/2 inhibitor AZD6244 (1 μM) for 72 h and cell viability were tested by CCK-8 assay. Cell viability of each treated group was compared with unpaired t-tests. *: P < 0.05. (C) X-370-resistant primary B-ALL cells were treated with X-370 in the presence of 1 μM AZD6244 or not for 72 h and phosphorylated Akt and Erk1/2 were then detected by Western blot.

Since PI3Kδ-dependent Erk1/2 phosphorylation was a critical predictor of the activity of X-370 in Raji cells, we further tested whether X-370 acted in the same manner in primary B-ALL cells. Indeed, both phosphorylated Akt and Erk1/2 dramatically decreased after treatment with low concentrations (< 1 μM) of X-370 in sensitive (IC_50_<1 μM) specimens. Even though X-370 was able to inhibit Akt phosphorylation in resistant (IC_50_>1 μM) samples, phosphorylated Erk1/2 remained unaffected (Figure 6A). Furthermore, co-treatment of AZD6244 with X-370 significantly enhanced activity against X-370-insensitive primary B-ALL cells (Figure 6B), and combination treatment was accompanied with decreased phosphorylation of Erk1/2 (Figure 6C). Taken together, these data demonstrated that X-370 significantly inhibited the viability of primary childhood B-ALL cells exhibiting PI3Kδ-dependent Erk1/2 signaling, and that PI3Kδ is a promising therapeutic target against childhood B-ALL. Combinatorial use of MEK1/2 inhibitor might be a rational strategy to overcome the resistance to PI3Kδ inhibitors in tumors demonstrating PI3K independent activation of the Erk1/2 pathway.

## DISCUSSION

The present study demonstrates that X-370 is a selective PI3Kδ inhibitor with potent activity against B-ALL cell lines and primary pediatric B-ALL cells. X-370 is distinguished by its structure and new interaction mode with PI3Kδ. Notably, X-370 inhibited Erk1/2 phosphorylation via an atypical PI3Kδ-PDK1-MEK1/2-Erk1/2 cascade in B-ALL cells. These results highlight a promising strategy for pediatric B-ALL therapy by targeting PI3Kδ. Furthermore, PI3Kδ-dependent Erk1/2 phosphorylation might be a pharmacodynamic biomarker to monitor the response to PI3Kδ inhibitors.

PI3Kδ-mediated signaling pathway has emerged as a central mechanism underlying the survival and expansion of various malignant B-cells. PI3Kδ is often hyper-activated in B-cell malignancies as a result of activation of the BCR, or due to mutations in PI3Kδ itself, as reported recently [34]. We found that X-370 potently blocks Akt phosphorylation in B-cell leukemia Raji and SU-DHL-6 cells at a concentration range similar to that required to inhibit the kinase activity of PI3Kδ, which is consistent with the previous studies of CAL-101 in CCRF-SB cells[12]. These results indicate that PI3K signaling is highly dependent on PI3Kδ activity in at least some B-cell leukemia cell types. X-370 potently inhibited the proliferation of a panel of B-cell leukemia cells. Furthermore, X-370 potently reduced the viability of 8/13 of the tested primary pediatric B-ALL cells at IC_50_s less than 1 μM with only 1/7 of specimen in non B-ALL cohorts was sensitive to X-370 treatment, further supporting the notion that PI3Kδ is a promising target for B-ALL therapy. On the other hand, we noticed that some primary B-ALL were highly tolerant of X-370, which highlights the heterogeneity of B-ALL.

As there is high variety in sensitivity to PI3Kδ inhibition among B-ALL cell lines and primary cells, identification of biomarkers capable of monitoring the efficacy of PI3Kδ inhibitors will be of great value. We found that inhibition of PI3Kδ led to inactivation of Erk1/2, indicating a PI3Kδ-dependent activation of Erk1/2 in B-ALL cells. Signaling dynamics are often influenced positively or negatively by an interplay between Ras-Raf-MEK-Erk and PI3K-Akt-mTOR pathways the two pathways [35-38]. The level of Gab1 in the cellular membrane fraction remained unchanged after X-370 treatment, indicating inactivation of Erk1/2 by PI3Kδ inhibition isn't due to the disruption of the feedback loop mediated by Gab1. Meanwhile, inhibition of PDK1 and MEK1/2 resulted in inactivation of Erk1/2, which is consistent with the previous report that PDK1 phosphorylates MEK1/2 biochemically and in cellular contexts [33]. Moreover, inhibition of PI3Kδ by X-370 abrogated the association between PDK1 and MEK1/2 suggesting that PDK1 phosphorylates MEK1/2 in a PI3Kδ-dependent way in B-ALL cells. This dependence highlights the importance of PI3Kδ in regulating both Akt-mTOR and MEK1/2-Erk1/2 pathways. We elucidated that X-370 inhibited Erk1/2 phosphorylation in B-ALL cells in an atypical PI3K-PDK1-MEK1/2-Erk1/2 cascade and it appears that inhibition of Erk1/2 phosphorylation may be utilized to monitor the efficacy of PI3Kδ inhibitors against B-ALL based on the following facts: first, X-370 failed to affect the phosphorylation status of Erk1/2 in Raji-R cells, which are resistant to X-370; second, constitutively active MEK DD partially rescued survival of Raji cells upon X-370 treatment; third, primary pediatric B-ALL cells displaying PI3Kδ-dependent Erk1/2 phosphorylation are more sensitive to X-370 (IC_50_ <1 μM), and *vice versa*; and finally, combination treatment of X-370 and AZD6244 significantly enhanced efficacy against primary pediatric B-ALL cells harboring PI3K-independent Erk1/2 phosphorylation. This crosstalk between PI3K and MEK1/2 underscores the important function of PI3K in initiation and progression of B-cell leukemia. Our data support the notion that PI3Kδ-dependent Erk1/2 phosphorylation might be a pharmacodynamic marker for response to PI3Kδ inhibitor in the therapy of benign pediatric B-ALL, a hypothesis that should be further studied and tested with more patient samples. On the other hand, combinatorial use with MEK1/2 inhibitors may be a useful strategy to improve the efficacy of PI3Kδ inhibitors for B-ALL therapy, as successfully shown in other experimental cancer models [39-42].

In summary, X-370 functions as a novel PI3Kδ inhibitor with promising activity against B-ALL cells displaying PI3Kδ-dependent Erk1/2 signaling via an atypical PI3K-PDK1-MEK1/2-Erk1/2 signaling cascade. These findings suggest the therapeutic benefit of PI3Kδ inhibitors for the treatment of pediatric B-ALL and provide insight to optimize therapeutic strategy based on PI3Kδ targeting.

## Materials and Methods

### Reagents and antibodies

X-370 were synthesized with a purity >99% by Xcovery (West Palm Beach, FL, USA) and AZ628, PLX4032, AZD6244, BX-912, MK-2206, Rapamycin, AZD8055 (Selleck, Houston, TX), BI-D1870 (Enzo Life Sciences, Farmingdale, NY) were dissolved in DMSO at 10 mM and stored at −20°C. Antibodies against Akt, Akt-pS473, Akt-pT308, S6k1, S6k1-pT389, Erk1/2, Erk1/2-pT202/Y204, p110δ and MEK1/2-pS217/S221 were from Cell Signaling (Cell Signaling, Danvers, MA). Antibodies against Gab1, CD19, MEK1 and PDK1 were from Eptomics (Hangzhou, China). Antibodies against β-Actin and GAPDH were from Sigma-Aldrich (Sigma-Aldrich, MO) and KangChen Bio-tech (Shanghai, China) respectively. IGF-1, MCP-1 and M-CSF were from R&D systems (Minneapolis, MN) and LPA was from Sigma-Aldrich (St. Louis, MO). Raji and SU-DHL-6 cells were obtained from ATCC (Manassas, VA). Raji-R cells were kindly provided by Yajun Guo (Shanghai Jiao Tong University, Shanghai, China) and MEF cells were generous gift from Jean Zhao (Dana-Farber Cancer Institute, Boston, MA). Rh30 cells were obtained from Dr. P.J. Houghton (St. Jude Children's Research Hospital, Memphis, TN, USA).

### Cell culture

SU-DHL-6, Raji, Rh30 (RPMI 1640 supplemented with 10% FBS), Raji-R (RPMI 1640 supplemented with 20% FBS) and MEF (DMEM supplemented with 10% FBS) cells were cultured in sub-confluence. Raji cells stably expressing MEKDD (Addgene plasmid 31202) or Luciferase (Addgene plasmid 25894) were established by lentivirus infection and selected with 1 μg/mL puromycin.

### *In vitro* kinase profiling

*In vitro* kinase profile assays were analyzed by the Kinase Profiler Service (Millipore, United Kingdom) following its guidelines. A GST-tagged human p110δ and regulatory full-length p85 were co-expressed in insect cell Sf9. Recombinant protein was extracted by affinity purification. The PI3K kinase activity was determined in the increasing concentration of ATP and X-370 with PI3-Kinase HTRF™ Assay kit (Millipore) as described previously [43].

### Docking stimulation

The crystal structures of PI3Kδ co-crystallized with ZSTK474 (PDB ID: 2WXL) and with IC87114 (PDB ID: 2WXE) were retrieved from RCSB Protein Data Bank[44]. The kinase domain of PI3Kδ was extracted as the receptor for molecular docking by Glide 5.0 (Schrödinger, LLC) with extra precision (XP). X-370 and CAL-101 were docked into 2WXL and 2WXE respectively. The docked ligand-protein complexs were presented by PyMOL 1.5 (Schrödinger, LLC).

### Cell viability assays

Cell viability was assessed using Cell Counting Kit (CCK-8, Dojindo, Japan) following the manufacturer's protocol and OD values were measured with a Spectramax M5 plate reader (Molecular Devices, Sunnyvale, CA)[45].

### Membrane protein separation and immunoprecipitation

Cell membrane fractions were separated with ProteoExtract® Native Membrane Protein Extraction Kit (EMD Millipore, Darmstadt, Germany). Immunoprecipitation was performed with a standard procedure. The final solution and total cell lysate were subjected to western blot.

### Flow cytometry assays

Apoptosis was measured with annexin V–fluorescein isothiocyanate (FITC) /Propidium iodide (PI) kit (KeyGEN Biotech, Nanjing, China) as previously described[46]. Fluorescence was acquired with a FACSCalibur instrument (BD Biosciences, CA) and analyzed using BD CellQuest Pro software.

### Primary B-ALL samples and X-370 sensitivity

Bone marrow was obtained from patients after provision of informed consent using guidelines approved by the Committee on the Use of Human Subjects in Research at Shanghai Jiaotong University or Shanghai Fudan University under a protocol approved by the Review Board. All patients examined in this study were diagnosed immunophenotypically with B-ALL and had not received any therapy before sample collection. All the samples were obtained during clinically indicated procedures. ALL B cells were isolated from freshly collected bone marrow with Ficoll density gradient centrifugation (Ficoll-Plaque Plus, GE Healthcare, WI). Enriched cells cultured in complete RPMI 1640 medium supplemented with 10% fetal bovine serum (Gibco, Grand Island, NY) were immediately seeded in 96-well plates in triplicate and treated with tested compounds for 72 h. Cell viability and phosphorylated Akt and Erk1/2 were then detected.

### Data analysis and statistics

For viability assays, results are shown as mean ±/+ SD of at least three experiments each. Student's paired or unpaired t-tests were employed for statistical comparison using GraphPad Prism 5. p-values less than 0.05 were considered as statistically significant and marked as *.

### Conflict of interest statement

Chris Liang is the Chief Scientific Officer in Xcovery and other authors declared no conflict of interest.

## SUPPLEMENTARY MATERIAL, FIGURES AND TABLES




