# Supplementary material for: Simultaneous targeting of PI3Kδ and a PI3Kδ-dependent MEK1/2-Erk1/2 pathway for therapy in pediatric B-cell acute lymphoblastic leukemia
Source: Oncotarget. 2014 Sep 26;5(21):10732–44. doi: 10.18632/oncotarget.2533 (PMC4279406; doi:10.18632/oncotarget.2533)
Supplement: Supplementary file 1 [file oncotarget-05-10732-s001.pdf]

Simultaneous targeting of PI3Kδ and a PI3Kδ-dependent MEK1/2-Erk1/2 pathway for therapy in pediatric B-cell acute lymphoblastic leukemia

Supplementary Material
Methods

Western blot analysis

Cells were treated as indicated conditions and then subjected to lysate to perform standard Western blot as described previously[1]. Briefly, whole-cell lysates and SDS-loading buffer diluted cell fractions were analyzed on SDS-PAGE gels. Transfer to nitrocellulose membranes, nonfat milk blocking, probing with indicated primary antibodies and the corresponding HRP-conjugated second antibodies. Chemiluminescence were performed and captured using ImageQuant LAS 4000 (GE healthcare Life Sciences, Piscataway, NJ, USA) and band intensity was measure both with ImageQuant™ TL (GE Healthcare Life Sciences) [2].

Caspase 3/7 activity assay

Raji cells were treated with or without X-370 at 1 µM, 5µM and 10 µM for 24, 48 and 72 hours, thenwere measured using the Caspase-Glo® 3/7 Assay (Promega, US) following the protocol and relative caspase-3/7 activity was normalized to cell number which DMSO control was the base line.

5-bromo-2'-deoxyuridine (BrdU) incorporation Assay

5-bromo-2'-deoxyuridine (BrdU) incorporation was performed using FITC BrdU Flow Kit (BD Pharmingen, San Diego, CA) according to the provided protocol.

Results

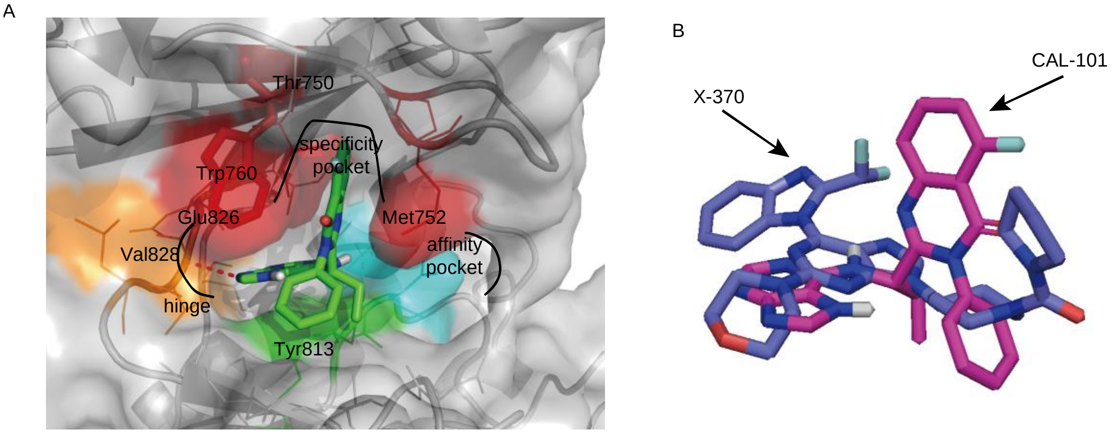

Figure S1;. CAL-101 docked to the crystal structure of PI3Kδ. (A) The model was generated based on the superimposition of 2X38 illustrating that the purine moiety establishes hydrogen bonds to the hinge residues Glu826 and Val828 and that the quinazolinone moiety is sandwiched into the induced hydrophobic specificity pocket between Trp760 and Met752. (B) Superposition of X-370 and CAL-101 to demonstrate their different modes of binding within the active site of p110δ.

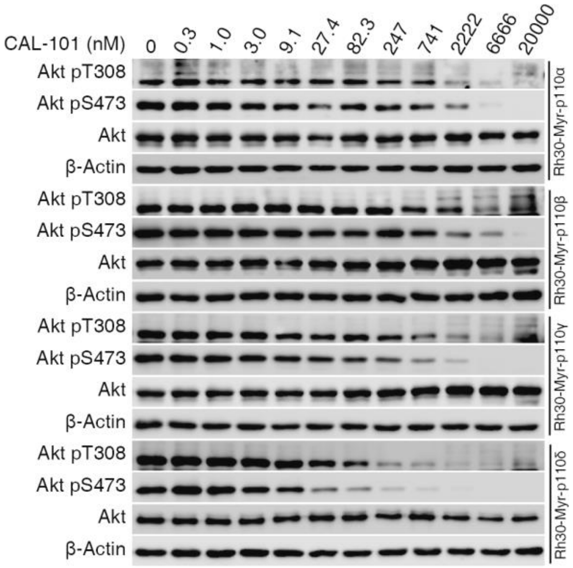

IC50 (μM) in Rh30-Myr-p110s

n=3	PI3Kα	PI3Kβ	PI3Kγ	PI3Kδ
Akt pT308	1.54	1.74	0.71	0.039
Akt pS473	1.39	0.39	0.15	0.012

Figure S2: CAL-101 is highly selective against PI3Kδ-mediated signaling at cellular level. The panel of isogenic Rh30-Myr-p110α, Rh30-Myr-p110β, Rh30-Myr-p110γ and Rh30-Myr-p110δ cells were cultured in serum-free medium for 12 h and then incubated with serially diluted CAL-101 for an additional 1 h. Cell lysates were probed with indicated antibodies. The IC50 values were calculated based on relative band intensity measured with ImageQuant™ TL (GE Healthcare Life Sciences, Piscataway, NJ). Data shown are mean values from three independent experiments.

Table S1: Inhibitory activity of X-370 against protein kinases

Kinase	activity (% of control)	Kinase	activity (% of control)	Kinase	activity (% of control)
Abl	130	IGF-1R, activated	120	PDGFR α	121
ALK	79	IKK α	95	PDK1	94
AMPK α1	101	IR, activated	111	Pim-1	88
ASK1	107	IRAK1	106	PKA	106
Aurora-A	73	Itk	106	PKB α	87
Axl	106	JAK2	109	PKC α	96
BTK	121	JNK1 α1	90	PKC μ	98
CaMKI	117	KDR	90	Plk1	100
CDK1/cyclinB	97	Lck activated	89	Ret	125
CHK1	74	LKB1	66	ROCK-I	93
CK1(y)	64	MAPK1	81	Ron	111
cKit	104	MAPKAP-K2	95	Rsk1	88
c-RAF	50	MEK1	109	SAPK2a(T106M)	102
DYRK2	102	MARK1	89	SGK	72
EGFR	90	Met	113	Src(1-530)	99
EphA1	99	mTOR	47	Syk	85
FAK	99	mTOR/FKBP12	48	TBK1	93
FGFR1	108	NEK11	95	Tie2	88
Flt1	99	p70S6K	88	TrkA	101
Fyn	97	PAK2	100		
GSK3 α	94	PEK	105		

X-370 possessed little inhibitory activity against a panel of 61 protein kinases representative of the human kinome at 10 μM. The relative kianse activity left in the presence of 10 μM were shown (Millipore KinaseProfiler Service).

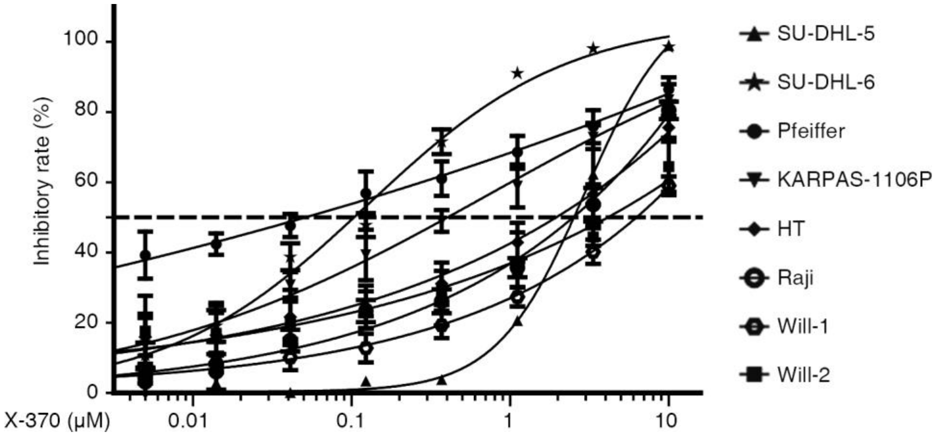

Figure S3: X-370 inhibited proliferation of human B cell leukemia cells. Cells were seeded in 96-well plate and treated with serial diluted X-370 for 72 h. cell viability was then assessed by CCK-8 reagent and inhibitory rate was calculated. IC50s ranged between 0.04 μM and 6.3 μM, showing the potent antiproliferative activity of X-370 against these cells.

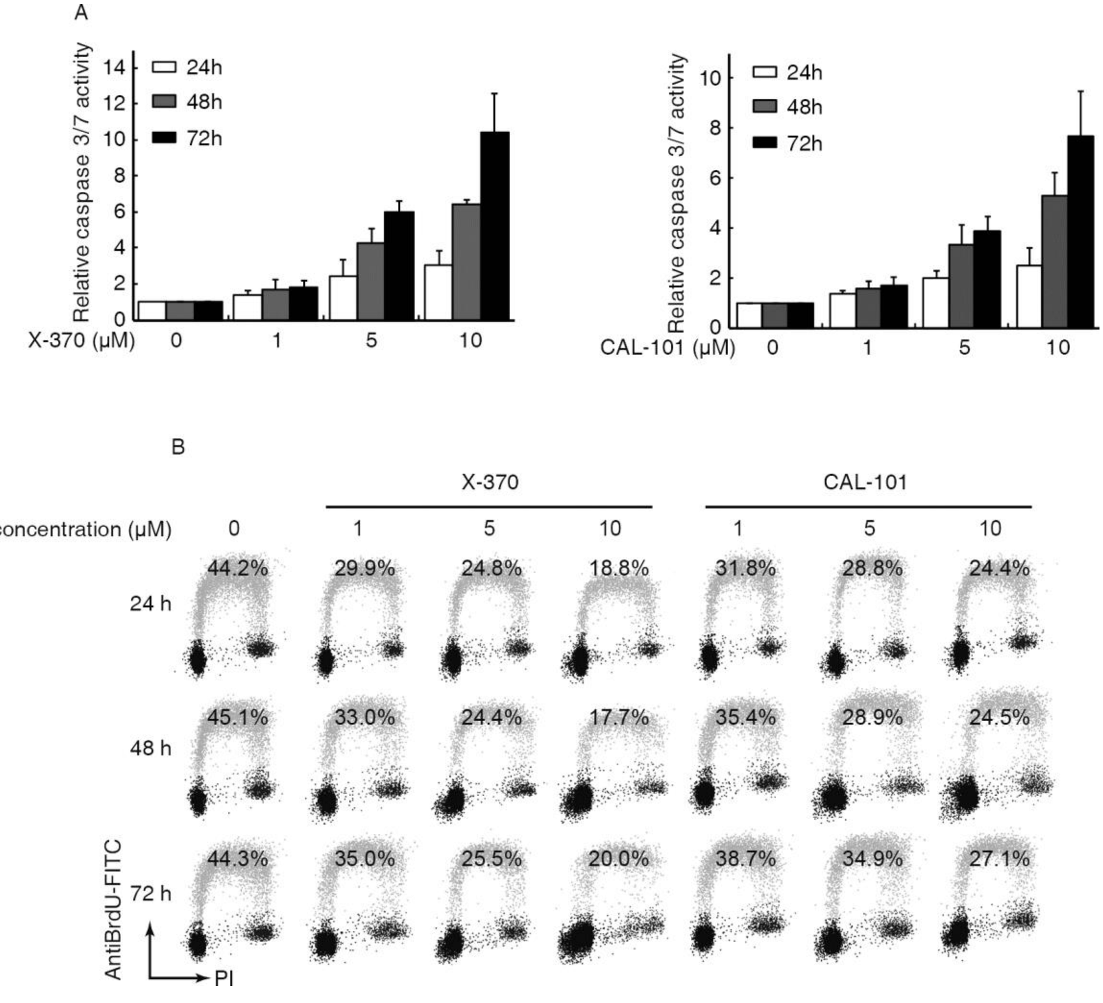

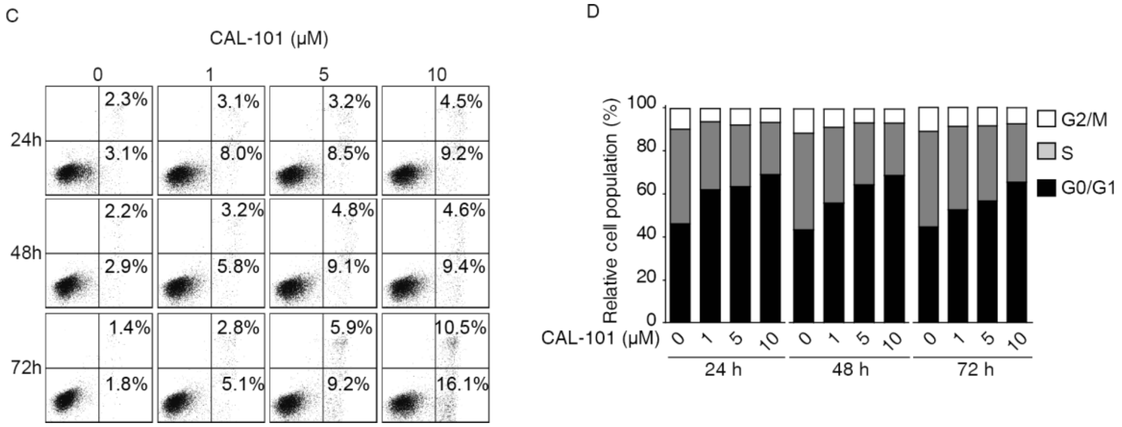

Figure S4: Cell effects of X-370 and CAL-101 treatment in Raji cells. Raji cells were treated with X-370 or CAL-101 at indicated concentrations for indicated times and then subjected to analyzing Caspase 3/7 activity (A) and BrdU incorporation (B). Caspase 3/7 activity slightly increased in dose- and time-dependent manners, while cell population in S phase decreased in a dose- but not time-dependent style and percentages of cells in S phase were labeled. Raji cells treated with CAL-101 for indicated times and at serial diluted concentrations were subjected to Annexin V-FITC/PI staining (C) and PI staining (D) and FACS analysis to show cell apoptosis and cell cycle distribution respectively.

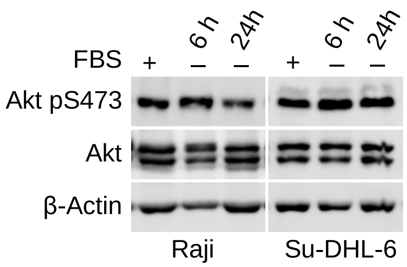

Figure S5: Raji and SU-DHL-6 cells showed constitutive activation of Akt when cultured in media either containing FBS or not. Raji and SU-DHL-6 cells were cultured in 10% FBS or in FBS-free medium for 6 h and 24 h respectively. Akt phosphorylation at Ser473 were unchanged, demostrating the constitutive PI3K activation.

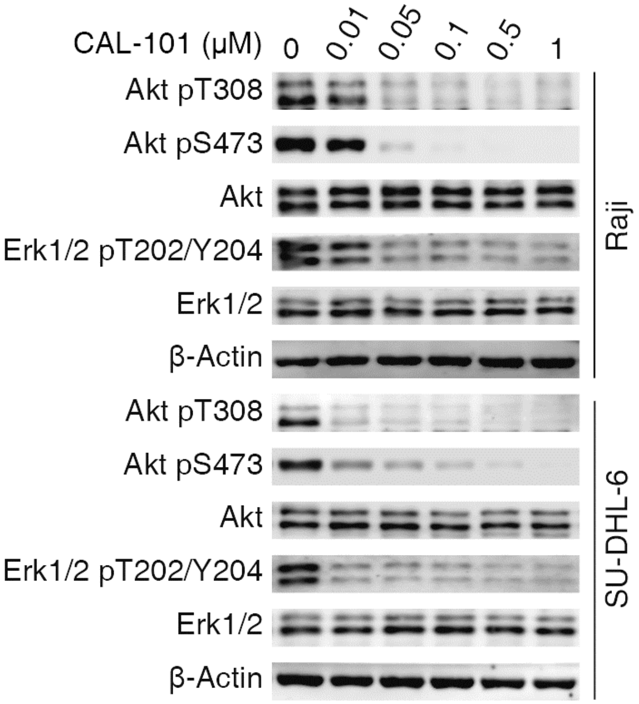

Figure S6: CAL-101 inhibited both Akt and Erk1/2 signaling in Raji and SU-DHL-6 cells. Raji and SU-DHL-6 cells were incubated with CAL-101 for an hour and then collected to perform Western blot, the phosphorylation of Akt and Erk1/2 were downregulated at low nanomolar concentrations.

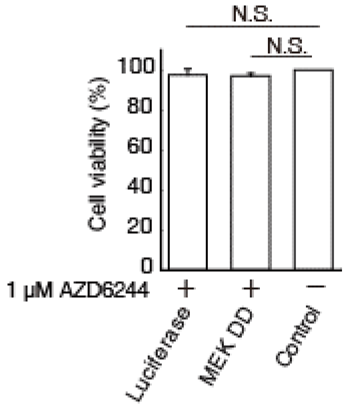

Figure S7: AZD6244 had little antiproliferative activity against Raji cells expressing MEK DD or luciferase. Raji cells expressing MEK DD or luciferase were incubated with 1 μM AZD6244 or DMSO control respectively for 72 h, and then cell viability were assessed by CCK-8 reagent. NS indicated no significance. Data were show as mean + SD of three independent experiments.

References

1. Li T, Wang J, Wang X, Yang N, Chen SM, Tong LJ*, et al.* WJD008, a dual phosphatidylinositol 3-kinase (PI3K)/mammalian target of rapamycin inhibitor, prevents PI3K signaling and inhibits the proliferation of transformed cells with oncogenic PI3K mutant. *The Journal of pharmacology and experimental therapeutics* 2010 Sep 1; **334**(3)**:** 830-838.

2. Wang X, Li JP, Yang Y, Ding J, Meng LH. A pharmacological model reveals biased dependency on PI3K isoforms for tumor cell growth. *Acta pharmacologica Sinica* 2013 Sep; **34**(9)**:** 1201-1207.
