# Supplementary material for: Simultaneous targeting of PI3Kδ and a PI3Kδ-dependent MEK1/2-Erk1/2 pathway for therapy in pediatric B-cell acute lymphoblastic leukemia
Source: Oncotarget. 2014 Sep 26;5(21):10732–44. doi: 10.18632/oncotarget.2533 (PMC4279406; doi:10.18632/oncotarget.2533)
Supplement: Supplementary file 2 [file oncotarget-05-10732-s002.pdf]

| No. | gender | age | IC50 (uM) | illness diagnoses | naive cells | Immunology | | genetics | fusion genes |
|---|---|---|---|---|---|---|---|---|---|
| 1 | female | 6y | 0.153 | ALL-L2 | 98% | B cells | cCD22 94.6%；CD10 36.3%；CD19 77.1%；CD22 64.2%；CD79a 37.1%；CD34 72.3%；Tdt 86.4%；cu 61.9%； | 46,XX | fusion genes free |
| 2 | female | 2y | >10 | ALL-M3 | 87.6%% | myelocytes | MPO 22.9%; CD33 91.7%; CD13 78.0%; CD117 57.9%; CD34 0%; HLA-DR 0%; CD9 68.6%; CD123 34.3%; CD38 78.7%; | 46,XX，t(15;17)(q22;q21)[10] | t(15;17)，PML-RARa |
| 3 | male | 8y | 0.056 | ALL-L2 | 80.40% | B cells | CD19 78.0%；CD79a 74.6%；CD10 52.3%；CD20 2.2%；TDT 35.6%；cu 8.2%；CD34 24%；HLA-DR 89.6%；CD9 92.6%；CD38 99.65； | 46,XY | fusion genes free |
| 4 | female | 6y | 0.011 | ALL-L2 | 93.60% | B cells | CD19 82.8%；CD79a 74.8%；CD10 99.5%；CD20 82.2%；CD21 77%；cu 12.4%；Tdt 97.6%；sIgM 0% | 45,XX,-8,add(9)(p12),del(20)(q11) [3]/ 46,XX [3] | fusion genes free |
| 5 | male | 3m | >10 | ALL-L2 | 94.60% | B cells | CD19 100.0%; CD79a 36.8%; CD22 88.8%; TDT 96.0%; CD13 47.5%; CD34 60.9%; HLA-DR 97.0%; CD38 99.7%; | 46,XY,t(4;11)(q21;q23) | t(4;11)(q21;q23) MLL-AF4 fusion |
| 6 | male | 2y | 0.263 | ALL-L2 | 97.20% | B cells | CD19 80.5%；CD79a 78%；CD10 97.5%；sIgM 0%；cu 6.5%；Tdt 97.3%；CD22 2.9%；CD20 7.4%；cCD22 56.2% | 46,XY | TEL-AML1 |
| 7 | male | 7m | >10 | ALL-L2 | 93.80% | B cells | CD19 99.3%; CD79a 96.7%;CD10 83.7%;CD34 0%;HLA-DR 98.9%;CD9 78.6%; CD38 99.1%; | 46,XY[6] | fusion genes free |
| 8 | female | 1y | 4.668 | ALL-L2 | 98.40% | B cells | CD19 99.0%; CD79a 68.5%;CD10 99.1%; CD22 82.7%; TdT 89.9%; CD64 26.8%; CD34 99.0%;HLA-DR 98.6%;CD9 99.1%; CD123 97.6%; CD66c 98.8%; CD38 64.9%; | ND | fusion genes free |
| 9 | male | 4y | 0.533 | ALL-L2 | 96.80% | B cells | CD19 97.9%; CD79a 84.1%; CD10 80.1%; CD22 78.1%; TdT 94.7%; CD20 35.0%; CD34 18.8%;HLA-DR 99.2%;CD9 99.4%; CD123 37.6%; CD66c 27.6%; CD38 98.6%; | 46,XY[15] | fusion genes free |
| 10 | female | 4y | 0.0015 | ALL-L2 | 97.60% | B cells | CD19 96.1%; CD79a 76.8%; CD10 97.9%; CD22 13.2%; TdT 97.6%; CD20 18.3%; CD34 74.2%;HLA-DR 97.5%;CD9 79.4%; CD123 5.8%; CD66c 0%; CD38 98.9%; | ND | TEL-AML1 |
| 11 | male | 5y | 0.569 | ALL-L2 | 92.80% | B cells | CD19 75.0%; CD79a 44.9%; CD10 87.0%; CD22 17.9%; TdT 90.0%; CD34 60.6%;HLA-DR 78.3%;CD9 35.4%; CD123 22.9%; CD66c 0%; CD38 67.2%; | 46,XY,del(20)(q11)/46,XY | TEL-AML1 |
| 12 | male | 12y | >10 | AML-M5 | 80.00% | myelocytes | CD33 76.9%; CD13 95.5%; MPO 18.9%; CD15 67.7%; CD117 96.3%; CD34 98.1%; HLA-DR 62.8%; CD123 76.4%; CD38 74.7%; CD133 22.4%; | ND | inv16, CBF-MYH11 |
| 13 | female | 3y | 0.17 | ALL-L2 | 91.00% | B cells | CD19 97.7%; CD79a 71.3%; CD10 85.6%; CD22 95.6%; TdT 96.1%; CD34 98.6%;HLA-DR 99.2%;CD9 99.0%; CD123 49.3%; CD66c 86.2%; CD38 96.5%; | ND | ND |
| 14 | male | 2y | >10 | ALL-L3 | 96.00% | T cells | CD7 99.1%; CD3 98.7%; CD4 88.8%; CD8 99.4%; CD1a 30.3%;  CD2 99.5%; CD5 99.2%; TDT 96.0%; CD34 12.2%; CD38 98.2%; | 46XY,-14,+21[2] | fusion genes free |
| 15 | male | 7y | 0.11 | ALL-M2a | 41.20% | myelocytes | CD7 85.1%; MPO 96.8%; CD33 37%; CD13 26.7%; CD64 67.3%; CD36 18.3%; CD15 75.5%; CD71 39.0%; CD117 91.2%; CD34 77.7%;HLA-DR 86.6%; CD123 35.3%; CD38 99.5%; CD56 73.9%; CD133 25.7%; | ND | ND |
| 16 | female | 5y | >10 | ALL-L1 | 73.60% | B cells | CD19 99.4%; CD79a 86.4%; CD22 60.7%; TDT 98.6%; CD34 95.1%; HLA-DR 75.5%; CD9 100.0%; CD123 45.8%; CD38 88.3%; CD56 13.8%; | ND | ND |
| 17 | female | 3y | 2 | ALL-L1 | 95.20% | B cells | CD19 74.9%; CD79a 51.2%; CD22 94.3%; CD10 95.4%; CD2 65.0%; CD34 98.6%; HLA-DR 96.9%; CD38 99.0%; | ND | ND |
| 18 | male | 8m | 5 | AML-M3 | 77.20% | myelocytes | CD4 53.8%; MPO 98.4%; CD33 99.0%; CD11b 38.3%; CD64 70.1%; CD15 94.1%; CD34 0.2%; HLA-DR 25.6%; CD9 22.0%; CD123 41.4%; CD66c 30.3%; CD38 87.2%; CD56 87.2%; CD133 10.9%; | 46,XY | fusion genes free |
| 19 | male | 8y | >10 | AML-M3 | 90.80% | myelocytes | CD33 99.3%; MPO 94.9%; CD13 83.5%; CD15 22.3%; CD71 22.0%; CD117 42.0%; CD123 74.3%; CD66c 63.6%; CD38 82.6% | ND | t(15;17)，PML-RARa |
| 20 | male | 8y | 7 | CML | 93.20% | myelocytes | CD33 99.0%; CD13 87.9%; HLA-DR 13.5%;CD11b 56.8%; CD10 55.4%; CD56 48%; CD15 98.0%; CD64 32.5%; MPO 92.1%; CD117 3.2%; CD34 1.8%; CD7 0.4%; | ND | t(9;22), BCR-ABL1 |
